# Statistical Modeling Reveals the Effect of Absolute Humidity on Dengue in Singapore

**DOI:** 10.1371/journal.pntd.0002805

**Published:** 2014-05-01

**Authors:** Hai-Yan Xu, Xiuju Fu, Lionel Kim Hock Lee, Stefan Ma, Kee Tai Goh, Jiancheng Wong, Mohamed Salahuddin Habibullah, Gary Kee Khoon Lee, Tian Kuay Lim, Paul Anantharajah Tambyah, Chin Leong Lim, Lee Ching Ng

**Affiliations:** 1 Institute of High Performance Computing, Singapore; 2 Lee Kong Chian School of Medicine, Nanyang Technological University, Singapore; 3 Ministry of Health, Singapore; 4 National Environment Agency, Singapore; 5 National University of Hospital, Singapore; 6 Singapore Sports Institute, Singapore; Centers for Disease Control and Prevention, United States of America

## Abstract

Weather factors are widely studied for their effects on indicating dengue incidence trends. However, these studies have been limited due to the complex epidemiology of dengue, which involves dynamic interplay of multiple factors such as herd immunity within a population, distinct serotypes of the virus, environmental factors and intervention programs. In this study, we investigate the impact of weather factors on dengue in Singapore, considering the disease epidemiology and profile of virus serotypes. A Poisson regression combined with Distributed Lag Non-linear Model (DLNM) was used to evaluate and compare the impact of weekly Absolute Humidity (AH) and other weather factors (mean temperature, minimum temperature, maximum temperature, rainfall, relative humidity and wind speed) on dengue incidence from 2001 to 2009. The same analysis was also performed on three sub-periods, defined by predominant circulating serotypes. The performance of DLNM regression models were then evaluated through the Akaike's Information Criterion. From the correlation and DLNM regression modeling analyses of the studied period, AH was found to be a better predictor for modeling dengue incidence than the other unique weather variables. Whilst mean temperature (MeanT) also showed significant correlation with dengue incidence, the relationship between AH or MeanT and dengue incidence, however, varied in the three sub-periods. Our results showed that AH had a more stable impact on dengue incidence than temperature when virological factors were taken into consideration. AH appeared to be the most consistent factor in modeling dengue incidence in Singapore. Considering the changes in dominant serotypes, the improvements in vector control programs and the inconsistent weather patterns observed in the sub-periods, the impact of weather on dengue is modulated by these other factors. Future studies on the impact of climate change on dengue need to take all the other contributing factors into consideration in order to make meaningful public policy recommendations.

## Author Summary

As dengue virus transmission is through a human-to-mosquito-to-human cycle, the influence of meteorological factors on dengue is likely to be associated with their impact on mosquito populations and behavior. Other than the influence of weather factors, the shift of dominant serotypes and pre-emptive measures taken against dengue vectors may possibly affect the dengue transmission trend. In this study, we investigate the impact of weather factors on dengue in tropical Singapore, taking into consideration the disease epidemiology and profile of virus serotypes. We found that absolute humidity, as a composite index of mean temperature and relative humidity, is a more stable and better predictor for modeling dengue incidence than the other unique weather variables when virological factors are taken into consideration. This research suggests that absolute humidity needs to be considered together with all the other contributing factors in order to make meaningful public policy recommendations for dengue control.

## Introduction

Dengue fever (DF) is the most common vector-borne viral disease in humans and is distributed worldwide, mainly in tropical and subtropical countries. In recent decades, dengue has been expanding globally possibly due to climate change [1] and highly intra and extra-country connectivity through traffic, commerce, and migration [2]. DF is caused by one of four distinct dengue virus serotypes (DEN 1–4). This viral infection has resulted in an estimated 50 million to 100 million annual cases of DF worldwide, with about 500,000 of these cases developing into life-threatening Dengue hemorrhagic fever (DHF)/Dengue shock syndrome (DSS) [2], .

In Singapore, which is a tropical island city state, DF is endemic, with year-round transmission observed. The integrated vector control program, implemented by the government, that started in the late 1960s resulted in a prolonged period of low dengue incidence [5]. The key strategy for dengue control in Singapore is to tackle the root of the problem, which is to deny Aedes mosquitoes the place to breed, i.e., source reduction [6], [7]. With a multi-pronged approach [6], [7], Singapore had adopted: 1) preventive surveillance and control, in which daily mosquito surveillance operations are conducted with the aid of the Geographical Information System; 2) public education and community involvement through working with construction sites, schools and community councils; 3) enforcement for carrying out intensive search and destroy operations at outdoor as well as indoor areas under legal laws upon notification of a dengue cluster; and 4) research for combating dengue disease including polymerase chain reaction, rapid antigen test kits, sequencing and bioinformatics, etc.

In addition to the preventive surveillance approaches, general practitioners and hospitals in Singapore are obliged to report probable dengue cases to the Ministry of Health and all reported dengue cases of DF/DHF are then confirmed by one or more laboratory tests including anti-dengue IgM antibody, enzyme linked immunosorbent assay (ELISA), and polymerase chain reactions (PCR). To our knowledge, there was no change in the notification process during the period studied in this work.

In Singapore, more than 80% of notified dengue cases were hospitalized [8]. Although under intensive dengue surveillance, we still experienced dengue hyperendemic in 2005 and in 2013 [9], with the number of laboratory confirmed cases reaching 14,209 cases (with 27 deaths) and 22101 cases (with 7 deaths) respectively. The re-emergence of hyperendemic may be due to low herd immunity, shift of dominant serotypes, high subclinical dengue infection and weather conditions etc. In an earlier report based on Singapore dengue data [10], it is estimated that only 1 out of 23 dengue cases are diagnosed and notified, which indicates a substantially high unreported dengue rate, i.e., a majority of dengue cases is either asymptomatic or subclinical but they are able to transmit dengue viruses to uninfected mosquitoes to trigger further infections. Other than the high subclinical cases possibly causing the dengue transmission to worsen, the tropical weather condition favors the year-round presence of Aedes mosquitoes, which is key in the dengue-human transmission chain. Thus, a better understanding on the association between weather and dengue incidence is important for a more proactive surveillance strategy of dengue control.

The impact of weather on dengue incidence has been widely studied [11], [12], [13], [14], [15], [16], [17], [18] as it is relatively easy to obtain basic meteorological data in dengue affected countries. Earlier studies have found many specific relationships between weather factors and dengue incidence. For example, the seasonality of dengue is well established for Thailand [19], [20], [21] and Vietnam [22], where dengue epidemic coincides with the rainy season. Malaysia also reported a strong seasonal pattern but its correlation to weather appears to be more complicated [23]. The number of dengue cases in Malaysia appears to be positively correlated with two to three month lag to the heavy rain in the first wet season of the year. For specific weather variables in Singapore, mean temperature and relative humidity were found to be the most important weather factors upon comparing models which considered long-term climate variability and linear lag effects of weather variables including temperature, humidity and rainfall [24]. In another study from Brazil [25], maximum temperature and minimum temperature were found to be the best predictors for the increased number of dengue cases.

In Singapore, a model consisting of lag effects of mean temperature and rainfall was built and applied to forecast the number of dengue cases over a 16 week period [15], [26], [27]. Mean temperature and relative humidity at a lag of 2 weeks and Niño Southern Oscillation Index at a lag of 5 weeks were found to have significant impact on dengue [24]. However, the effect of absolute humidity on dengue incidence, which reflects the combined impact of temperature and relative humidity, has not been well described. In addition to weather, the impact of the dynamics of circulation of dengue virus serotypes on dengue epidemiology has been well documented [28]. Infection with one serotype confers life-long immunity to that particular serotype [29], [30]. Some studies have also reported a time-lagged correlation between dengue virus serotype dynamics and disease incidence rates [31]. The variation of dominant serotypes needs to be taken into account in studies of environmental factors on dengue incidence.

In this study, we modeled and compared the effect of absolute humidity with the effect of temperatures (maximum, minimum, mean), relative humidity, rainfall and wind speed on dengue in Singapore from 2001 to 2009. The model used is a distributed lag non-linear model, i.e., an over-dispersed Poisson model with regressions on autocorrelation, lagged effect of weather factors, population sizes and dengue trends. The model is further refined by comparing the impact of weather variables in sub-periods divided based on the dominant circulating dengue serotypes. The model selection criterion applied in this study is the Quasi Akaike's Information Criterion.

## Methods

### Study area

Singapore is a tropical island city state with approximately 710.2 km^2^ land area. The average size of the total population over the years, from 2001 to 2009, is about 4.41 million (Department of Statistics, 2013). The mean temperature ranges from 25.2°C to 30.3°C, with the maximum daily temperature and maximum daily rainfall reaching up to 34.5°C and 479.7 mm respectively.

A vector control program in the 1960s to 1980s had successfully prevented dengue outbreaks for two decades since 1973, with less than 1,000 reported cases per year [5]. However, since 1989, Singapore has observed increased notifications of dengue infection despite a low *Aedes* house index of less than 1%. The factors contributing to the re-emergence includes an increase in human population and density, increases in cross border and in country travel and low herd immunity, resulting from low transmission in the prior decade [5]. The most recent large outbreaks occurred in 2005 [32] and 2013 raise more concern on dengue spread in Singapore.

### Data collection

Weekly notified DF/DHF cases in Singapore from 2001–2009 were retrieved from the Weekly Infectious Diseases Bulletin [9] of the Singapore Ministry of Health. The human population data used was based on the mid-year Singapore total population data obtained from the Singapore Department of Statistics [33].

Whilst all four dengue serotypes have mostly been detected in Singapore, typically there is one predominant circulating serotype, with switches in predominance associated with the outbreaks (Table 1). The dominant serotype was defined as one that causes more than 50% of cases sampled. The estimated proportion of each viral serotype was obtained from the Singapore Communicable Diseases Surveillance reports [34] of Singapore Ministry of Health. DEN-2 was the dominant circulating serotype in the years 2001–2003, DEN-1 in 2004–2006 and DEN-2 in 2007 to 2009.

**Table 1:** Dengue serotype distribution in Singapore from 2001 to 2009.

	2001	2002	2003	2004	2005	2006	2007	2008	2009
Dengue cases	2372	3945	4788	9459	14,209	3127	8826	7031	4497
DEN-1	7%	30.7%	9.2%	**67%**	**67.4%**	**79%**	5.7%	21.9%	18.1%
DEN-2	**89%**	**53.8%**	**80.5%**	27.6%	8.7%	5.9%	**78.7%**	**57.4%**	**52.1%**
DEN-3	0	3.9%	4.6%	2.4%	17.9%	6.4%	4.1%	8.7%	18.7%
DEN-4	13%	11.6%	5.7%	3%	0.6%	3.7%	0.6%	0.9%	6.6%
Indeterminate	-	-	-	-	5.4%	5%	10.9%	11.1%	4.6%

**Figure 1:**
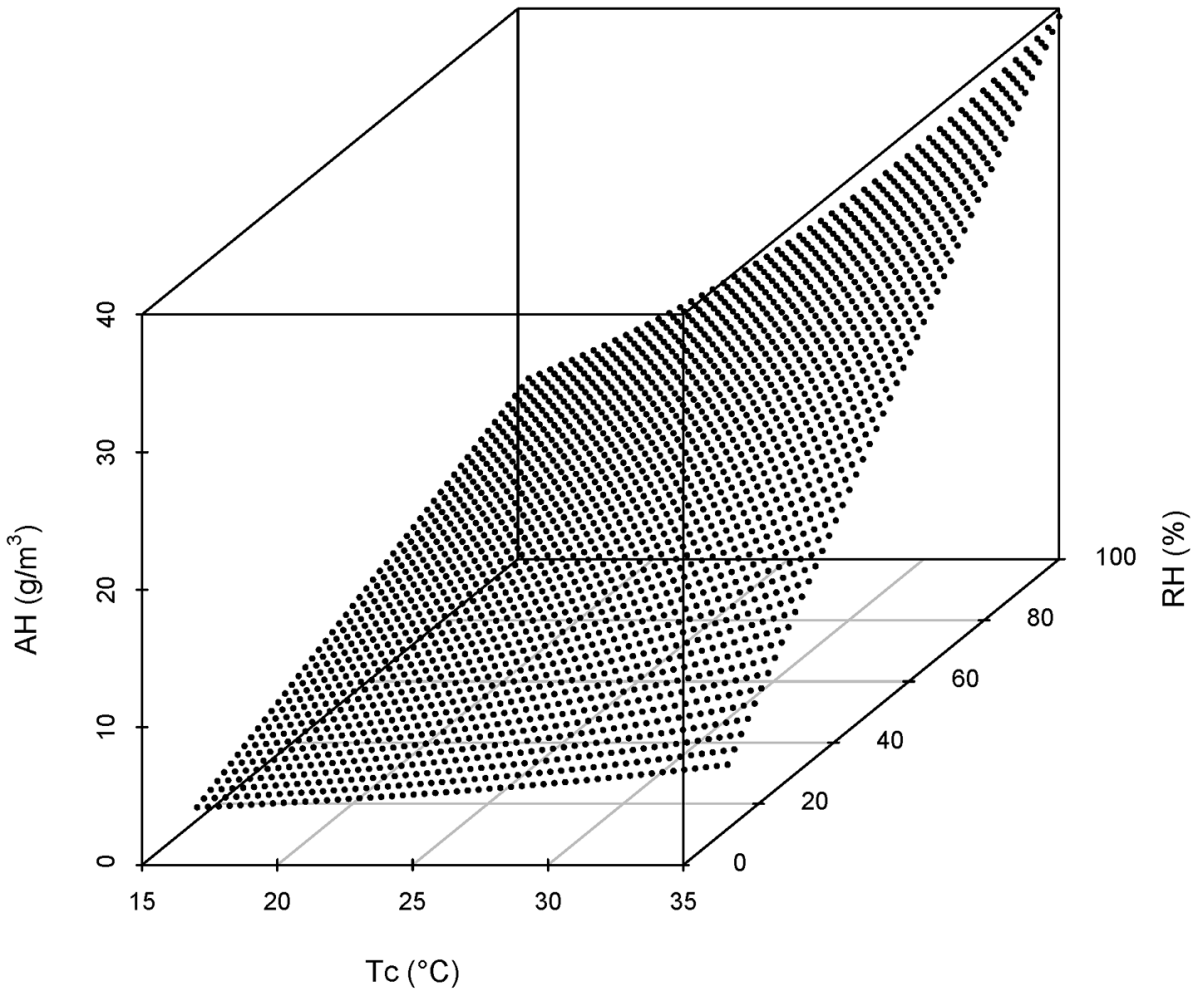
Scatter plot of AH v.s. Tc and RH ((Eq. 1)).

Weather data including Mean temperature (MeanT, °C), Minimum temperature (MinT, °C), Maximum temperature (MaxT, °C), Rainfall (Rain, mm), Relative humidity (RH, %) and Wind speed (WindS, m/s) were obtained from the National Environment Agency, Singapore. Absolute humidity (AH, g/m^3^), which is the mass of water in a unit volume of air, was estimated through dry bulb temperature and relative humidity using the approximated equation, assuming standard atmospheric pressure [35]:
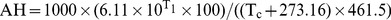
(1)where T_c_ is the dry bulb temperature (in our studies, T_c_ is the daily mean temperature), and
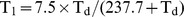
where T_d_ is the dew point temperature. T_d_ is approximated from the equation below, based on dry bulb temperature and relative humidity:
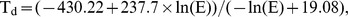
where 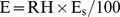, 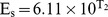 and 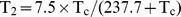. Weekly weather data were calculated by averaging the daily weather values over each week. The relationship between AH, T_c_ and RH is presented in Figure 1.

### Statistical analysis

**Figure 2:**
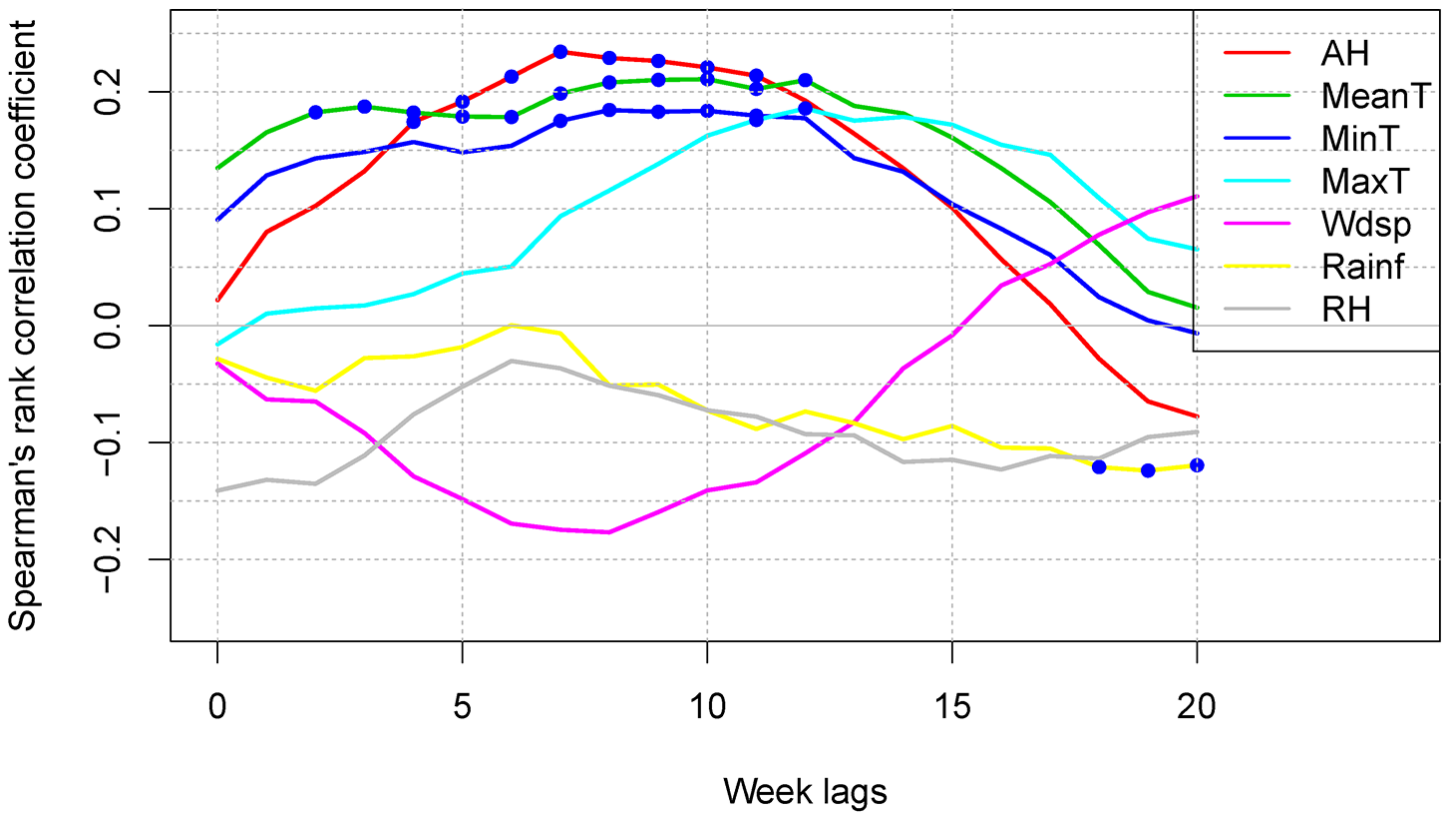
Time-lagged cross-correlation of dengue incidence and each weather variable (0 to 20-weeks lag). Significant correlation coefficients with p-value<0.05 are in solid circles.

Spearman rank correlation tests were then applied to assess the association between weekly dengue cases and weather factors for a range of time lags – from 0 to 20 weeks, over the whole study period (from 2001 to 2009) (see Figure 2). As the number of dengue incidence is a Poisson count data, it is thus not feasible to check how it is linearly related to weather factors. As such, Spearman rank correlation is usually chosen as it is designed to assess how well two variables are monotonically related even if their relationship is not linear [36]. As autocorrelation was detected in each time series, it would not be appropriate to calculate p-values of the correlation coefficients by traditional methods. Therefore, the p-values were calculated through Adaptive Wavelet-Based Bootstrapping [37] with a sample size of 5000. This was implemented in R software (version 3.0.2; package ‘wmtsa’). In this study, the p-value of the correlation coefficients between every two time series was calculated using this method.

Furthermore, the associations between each weather predictor and the risk of dengue were modeled. The number of observed dengue cases, 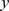, at week 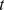, was assumed to follow an over dispersed Poisson distribution [38] with mean 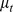. The effect of weather variable 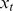 on 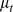 was described by a Distributed Lag Non-linear Model (DLNM) [39], [40] given as follows:
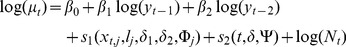
(2)where 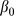 is the intercept, 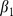 and 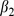 are coefficients of the auto-regression terms, 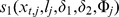 is a function to denote smoothed relationships between 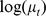 and a single weather factor 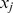 (i.e., MinT, MeanT, MaxT, Wdsp, Rainf, RH or AH) with a maximum lag number of 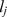, which enables to include the lag effect of predictors into the model. The nonlinear effect of weather factor 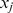 was described by a natural cubic spline (ns) smoothing function with 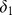 degrees of freedom (df) and knots at equally spaced quantiles, while the lag effect of 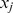 was described by an ns smoothing function with df of 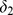. 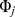 is the corresponding coefficients vector. 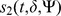 is an ns smoothing function with df of 1 per year applied to fit the long-term trend of dengue incidence. Here, the df, 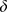 = 9 and 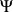 is the corresponding coefficients vector. 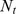 is the mid-year population size of Singapore and 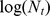 is the offset term. Besides the DLNM, the single lag effect of each weather factor was also investigated. When considering the effect of weather factor 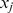 at lag 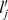, 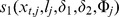 was replaced by 
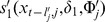
 in Eq. 1 with 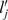 being the lag number, and 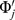 being the coefficients vector, i.e., the effect of 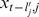 was modeled by an ns function with df of 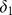.

In order to reflect the goodness-of-fit, Quasi Akaike's Information Criterion (QAIC) was used with a smaller QAIC implying a better fit [40], [41]. QAIC is given by
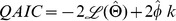
(3)where L is the log-likelihood of the fitted model with parameters 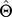 (in Eq.2, 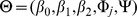) and 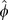 (i.e., the estimated overdispersion parameter), whereas k is the number of parameters. In 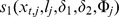 (Eq.2), 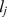 was selected from 0 to 20 weeks [15]. The df (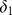) of each 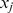 was selected from 1 to 5, while the df (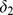) of lag was selected from 1 to 3. Higher df implies higher flexibility, but may introduce over-fitting. The selection criterion was QAIC and model flexibility. For the space of each weather variable, QAIC indicated 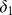 = 4 or 5 for all weather variables; whilst for the lag dimension, QAIC indicated 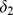 = 2 or 3. In this article, we adopted 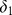 = 4 and 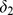 = 3. The analyses were performed in R software (version 2.13.2; package ‘dlnm’; R Development Core Team, 2011) [42]. We first investigated the maximum lag considering the overall effect of each weather variable on dengue incidence for the whole period. Once the best model was established based on the smallest QAIC, the model was further studied and evaluated for both the entire studied period and the three distinct sub-periods based on the predominant circulating serotypes.

## Results

### Absolute humidity-Relative humidity-Temperature relationships

We found that Absolute humidity (AH) was positively correlated with Relative humidity (RH) and Temperature (see (Eq. 1 and Figure 1)). The correlation coefficient between AH and RH is 0.21, whilst the correlation between AH and mean temperature is 0.54. A higher RH or a higher temperature was associated with a higher AH. However, the correlation between MeanT and RH was negative (the correlation coefficient is −0.71). Therefore, as a composite index of MeanT and RH, the impact of AH on dengue incidence was studied further.

### Whole period analysis

The Spearman rank correlation analysis, using time lagged weather data (0–20 weeks), showed that temperature (MeanT, MaxT, MinT), absolute humidity and rainfall exhibited significant association with dengue incidence. On the other hand, no significant relationship was observed between dengue and wind speed, and relative humidity. The correlation between AH and dengue incidence was the highest (its correlation coefficient was 0.234 with p-value<0.05 at a 7-week lag) among all the studied weather variables (see Figure 2). The second highest correlation was between MeanT and dengue, with the lag period of 12 weeks and a corresponding correlation coefficient of 0.211 with p-value<0.05. The correlation between rainfall and dengue incidence is, although significant, numerically quite small, about less than 0.15.

**Figure 3:**
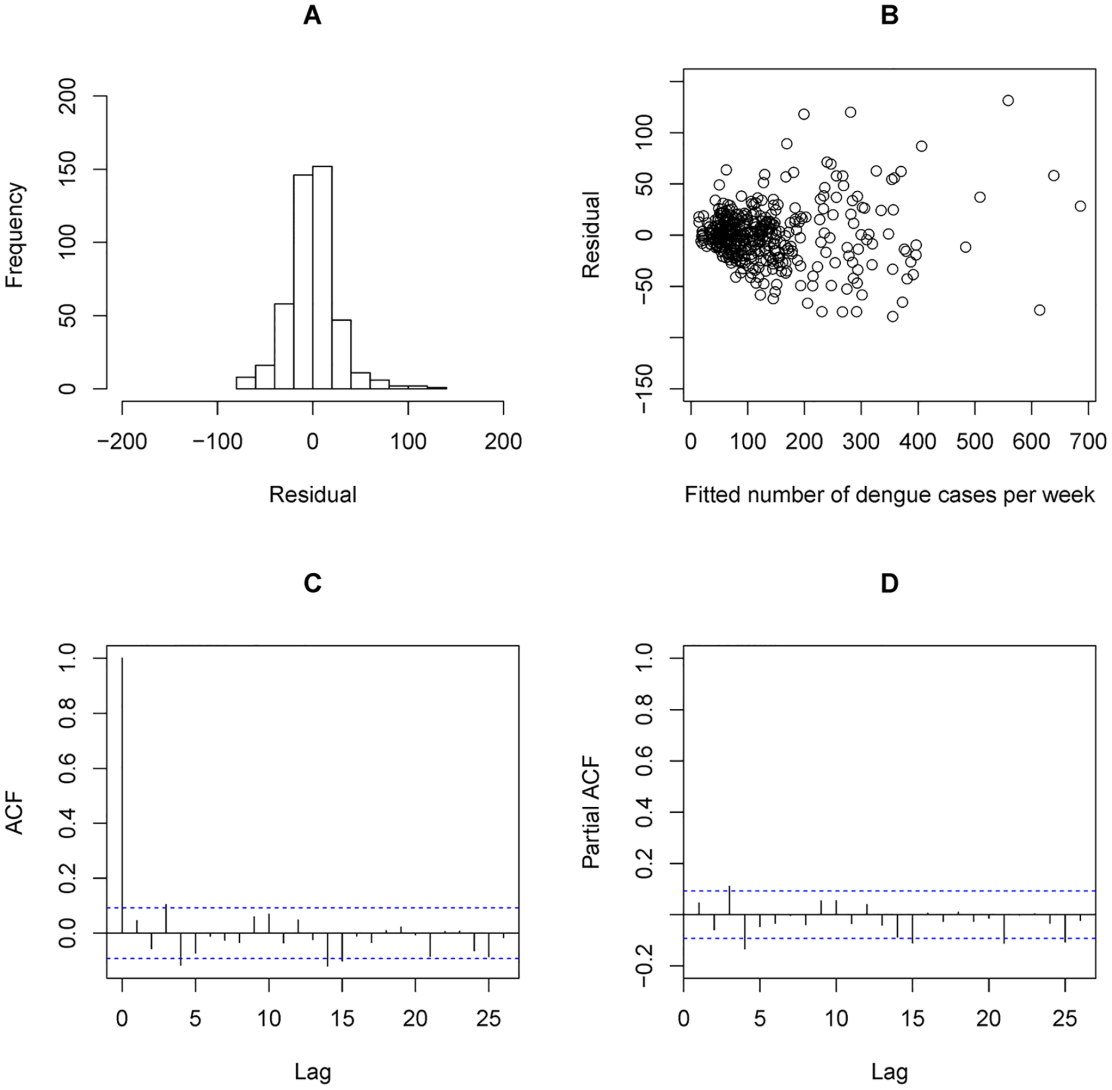
Residual analysis for DLNM-AH model. A: Residual histogram; B: Residual v.s. Number of dengue cases per week; C: Residual autocorrelation function; D: Residual partial autocorrelation function.

It was also observed that AH was associated with the smallest QAIC values, among all weather predictors in both single and distributed lag models (see Table 2). The best single lag effect of AH was 1 week, after adjustment for the impact of previous dengue incidence. When considering the cumulative lag effect of AH, a 0–16 weeks lag of AH showed the best fitting performance. Residual analysis is shown in Figure 3. The smaller the fitted number of dengue cases was, the less the variability of the residual values would be seen (Figure 3B). This supported our statement that overdispersion existed in the distribution of dengue. Autocorrelation function and partial autocorrelation function of residuals (Figure 3C & Figure 3D) demonstrated the independence of the residuals, implying that autocorrelation of the dengue cases has been explained by the DLNM-AH model.

**Table 2:** QAIC based on best lag number for each weather predictor considering DLNM.

Weather variables		MinT	MeanT	MaxT	Wdsp	RH	Rainf	AH
Single lag model	Best single lag	1	9	18	4	1	18	1
	QAIC	2530.02	2538.83	2441.76	2550.65	2534.39	2438.50	**2434.43**
Distributed lag model	Best maximum lag	19	9	19	12	11	19	16
	QAIC	2382.54	2435.62	2347.712	2540.207	2353.09	2329.184	**2231.25**

**Figure 4:**
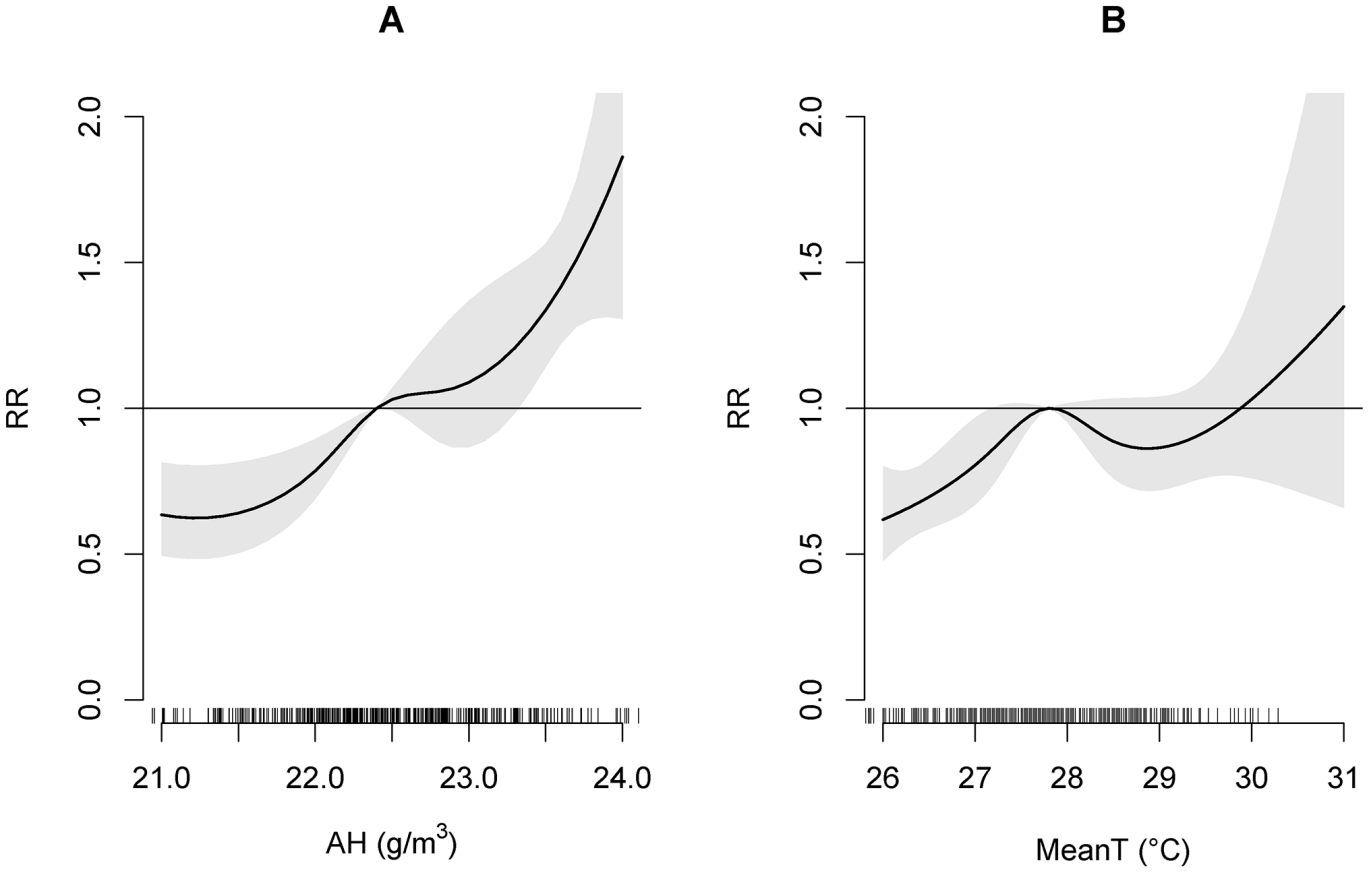
Effect of AH and MeanT on relative risk (RR) of dengue incidence. A: RR curve shows overall cumulative effect of AH (with the maximum lag number up to 16 weeks) on dengue incidence with reference value of AH being 22.4 g/m3 and 95% CI of fitted RR shown in the grey region; B: RR curve shows overall cumulative effect of MeanT (with the maximum lag number up to 9 weeks) on dengue incidence with reference value of MeanT being 27.8°C and 95% CI of fitted RR shown in the grey region.

Summing up each single lag effect from 0 to16 weeks, the 17-week overall effect of AH on relative risk of dengue incidence for the full period is shown in Figure 4A. It can be seen that a higher AH was associated with a higher dengue incidence. It is important to note that that the relative risk here is the ratio of the probability of dengue incidence occurring at a certain value of a weather variable to the probability of the event occurring at a reference value of the same weather variable. The change of reference points may affect the width of confidence interval, but it will not affect the RR curve itself. In some research work, mean was chosen as reference [43], while the point of overall minimum mortality was chosen as the reference in some other work [40]. Here, the reference value of AH is 22.4 g/m3, which is both mean and median of AH during the studied period.

**Figure 5:**
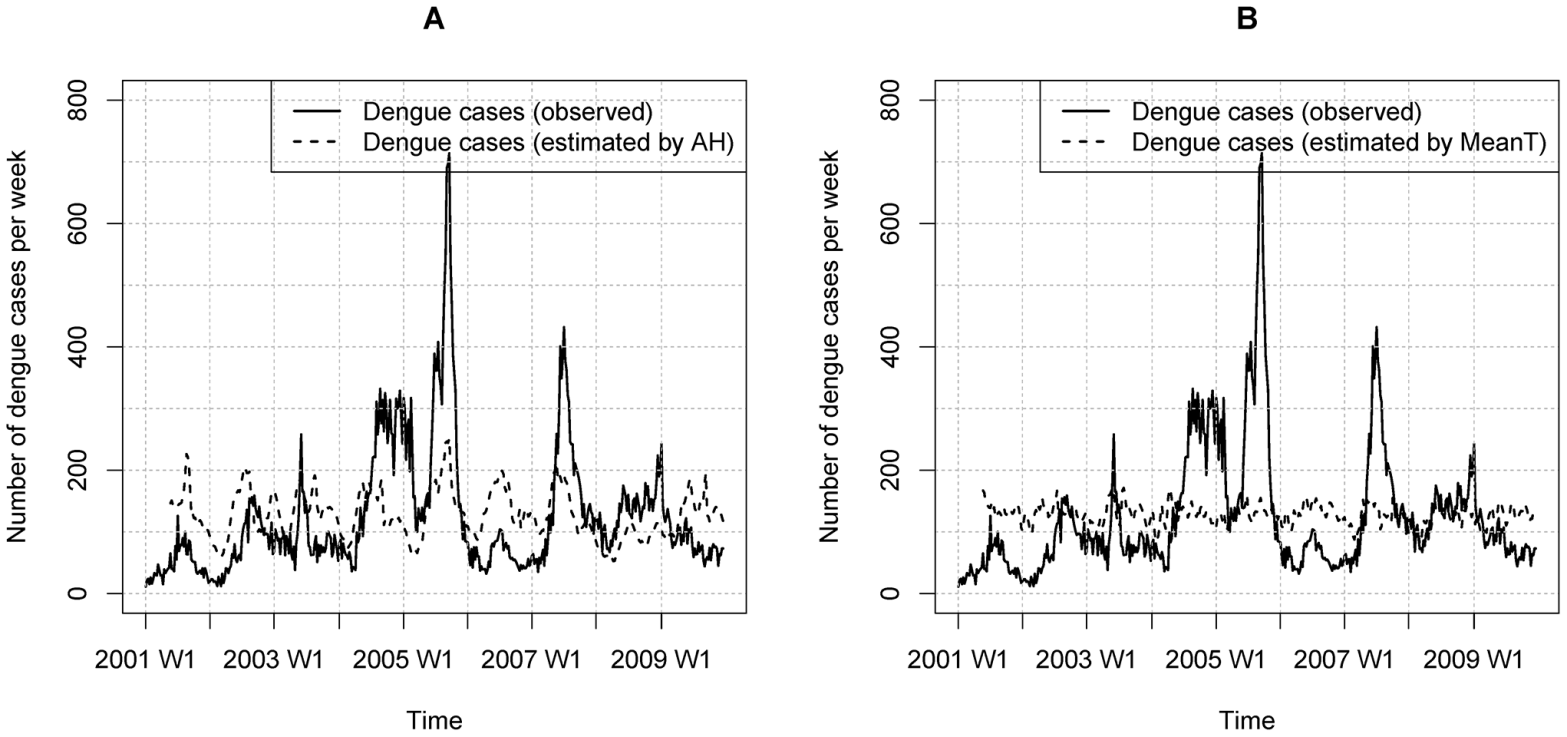
Weekly counts of dengue cases from 2001–2009. A: Observed dengue cases and number of fitted dengue cases estimated by the AH term in the DLNM model; B: observed dengue cases and number of fitted dengue cases estimated by the MeanT term.

The estimated weekly dengue incidence, using only the AH term (i.e., exp(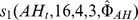), see Eq. 2) is shown in Figure 5A. The correlation coefficient between the estimated dengue and observed dengue cases is 0.374 (p-value<0.01), which shows a moderate positive relationship. It can be clearly seen that the peaks of AH and dengue incidence are very well synchronized.

**Figure 6:**
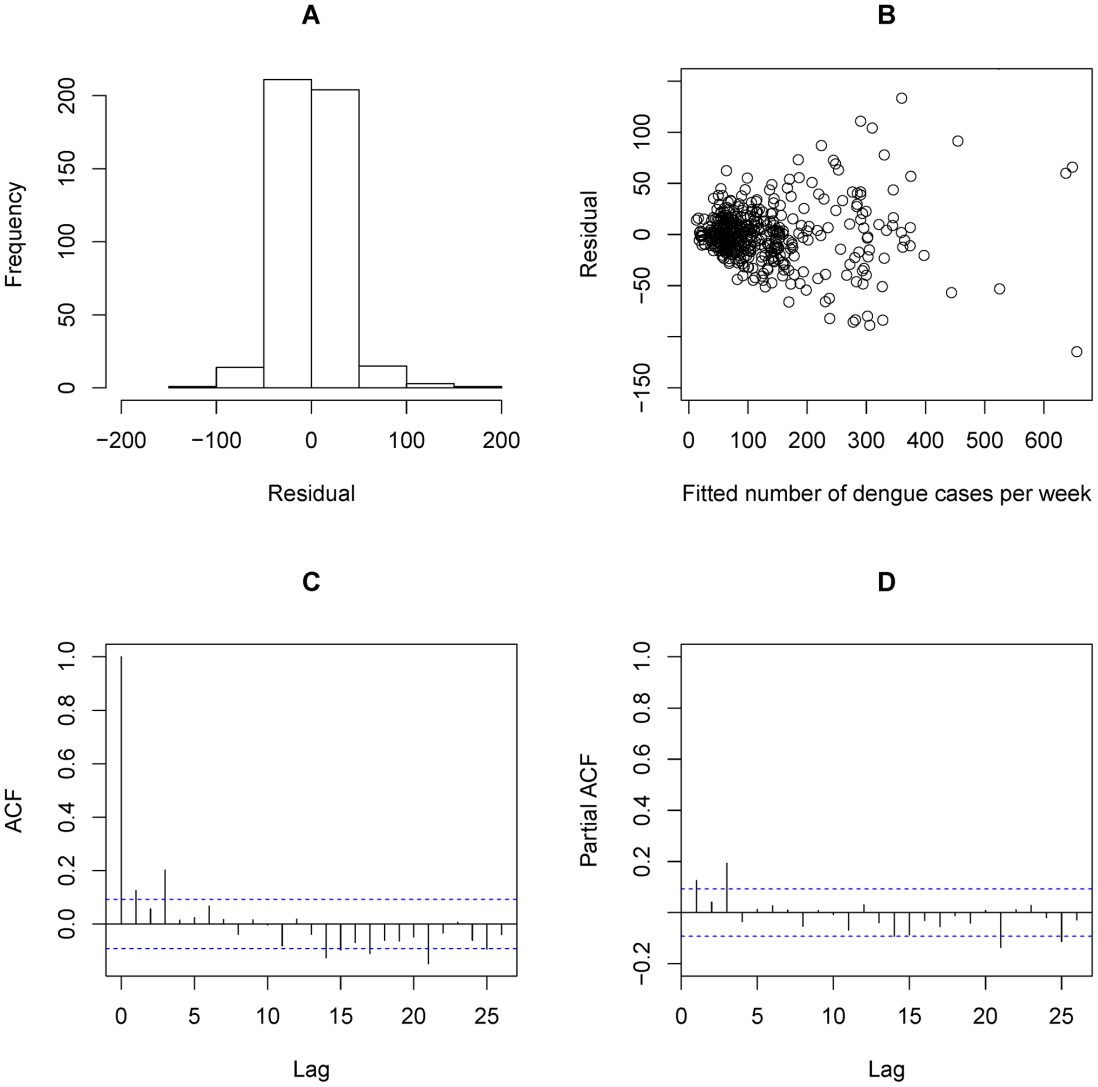
Residual analysis for DLNM-MeanT model. A: Residual histogram; B: Residual v.s. Number of dengue cases per week; C: Residual autocorrelation function; D: Residual partial autocorrelation function.

As MeanT has been used as an indicator by National Environment Agency (NEA) of Singapore for dengue surveillance in recent years [44], we also modeled MeanT's impact on dengue incidence and compared it with the impact of AH. Based on our model analysis, the longest lag that best reflects the effect of MeanT on dengue is 9 weeks. Residual analysis is shown in Figure 6. Similar phenomena were detected in the residuals compared with the residuals of the DLNM-AH model. Nevertheless, slightly higher values were detected in autocorrelation function and partial autocorrelation function of residuals (Figure 6C & Figure 6D).

The effect of 0–9 weeks lag of MeanT for the full period is shown in Figure 4B. In general, it can be seen that a higher MeanT is associated with a higher risk of dengue incidence but this observed relationship does not hold true when the MeanT is higher than 27.8°C. The estimated number of weekly dengue cases using the MeanT term, described in Eq. 1, is shown in Figure 5B, which showed that the correlation coefficient between the estimated dengue and the observed dengue cases is only 0.150.

### Sub-period analysis

**Figure 7:**
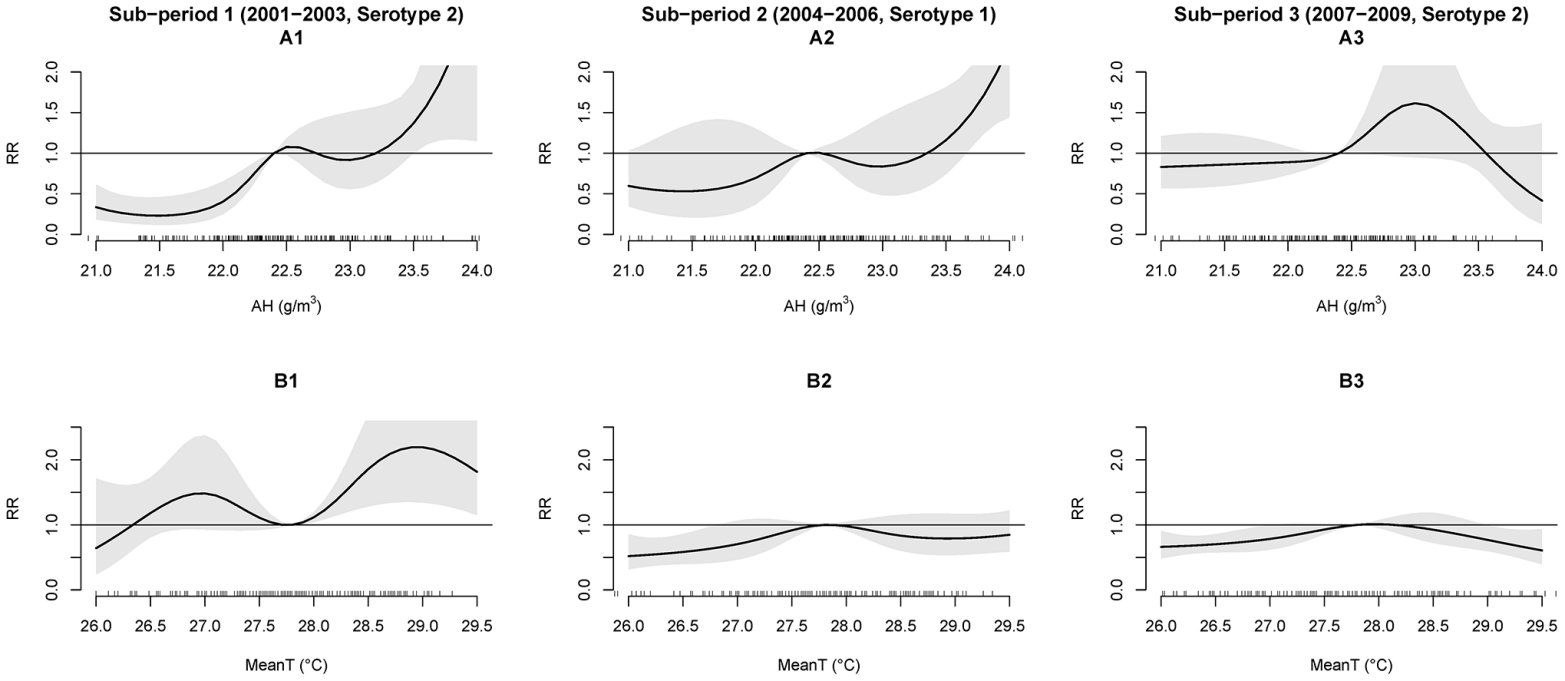
Effect of AH and MeanT on RR of dengue incidence obtained from the distributed lag model for each sub-period. A1–A3: Effect of 0–16 weeks lag of AH; B1–B3: Effect of 0–9 weeks lag of MeanT. The grey region indicates 95% CI of fitted RR. Reference AH = 22.4 g/m3 and MeanT = 27.8°C.

In addition to studying the pattern for the entire period (2001–2009), analyses were also carried out on the three distinct sub-periods, namely, 2001–2003 (sub-period 1, DENV2), 2004–2006 (sub-period 2, DENV1), and 2007–2009 (sub-period 3, DENV2). The aim is to evaluate the coupling effect of weather factors as well as the impact of the dominant serotypes in each period. The overall effects of AH on dengue incidence in each sub-period are presented in Figure 7(A1 to A3). In sub-period 1 and sub-period 2, the impact of AH on dengue incidence was found to be similar to that observed in the whole period, i.e. increasing the AH generally increased the risk of dengue incidence. However, in sub-period 3, it can be seen that the effect of AH on dengue was not significant.

The effect of 0–9 weeks lag of MeanT for each sub-period is shown in Figure 7(B1 to B3). It can be seen that the impact of MeanT on dengue incidence in the three sub-periods was not consistent across the three sub-periods or with the pattern observed during the whole period. In sub-period 1, the impact of MeanT on dengue was not significant when MeanT was less than 27.8°C; whilst in sub-period 2, this effect turned to be not significant when MeanT was higher than 27.8°C. Interestingly, the effect of MeanT in sub-period 3 was an inverse U curve, as shown in Figure 7(B3).

## Discussion

In general, rain, temperature and relative humidity had been the most common weather variables associated with dengue incidence and outbreaks [24], [45], [46]. The influence of these meteorological factors on dengue is likely to be associated with their impact on mosquito populations and behavior [47]. Rain provides more breeding habitats and opportunities for proliferation in the environment. There is also compelling evidence supporting the hypothesis that mosquito oviposition, development from mosquito larva to adult, biting rate and virus replication rate in mosquito are strongly enhanced at raised ambient temperatures [48], [49]. The hatch percentage for *Ae aegypti* eggs was also found to increase with the increase in relative humidity in Texas [50].

However, in our study, it was observed that there is no significant relationship between RH and dengue (see Figure 2). On the other hand, we found that temperature is positively correlated with the count of dengue cases, although temperature is negatively correlated with relative humidity. Hence, we further studied the relationship between AH and dengue in this work with the consideration that AH measures absolute moisture in the ambient air as a composite factor of mean temperature and relative humidity.

To reflect the influence of absolute moisture in the ambient air on dengue incidence, we explored the cross-correlation of dengue incidence with absolute humidity and found that it had the best correlation with dengue cases in Singapore among the major meteorological variables. Furthermore, as indicated by the DLNM-AH model, a moderate positive correlation between dengue and its estimation using only the AH term (correlation coefficient is 0.374, p<0.01) was obtained. This correlation coefficient is relatively high compared with other weather factors. Besides the significant correlation coefficient, it was also noted that the peaks of absolute humidity were well synchronized with dengue peaks. Although MeanT is being used for risk assessment of dengue by the authorities [44], our modeling results suggests that AH may be a better indicator to predict dengue incidence, as demonstrated by the RR curves and the higher correlation coefficient when compared to MeanT.

Interestingly, rainfall, which had been found to be associated with dengue in many places, did not seem to have much bearing on dengue cases in Singapore. This is perhaps consistent with the findings of the National Environment Agency which claimed that typically about 70% of breeding habitats of *Ae aegypti* were associated with homes and the most common breeding habitats were indoor ornamental containers and household items where the impact of rainfall is likely to be limited.

In our study, the effect of AH on dengue was found to have an optimal maximum lag of 16 weeks, an interval which is consistent with an earlier study [15], [26]. The non-linear lag effect of weather predictors on dengue incidence has also been reported in many studies [15], [26], [45]. The lagged effect of dengue incidence could account for the length of life cycle as well as the host-vector-pathogen transmission cycle of vectors [15].

MeanT is being used for dengue surveillance in recent years [44] in Singapore. Following our studies, when evaluating over the whole studied period and sub-period 2 and 3, no significant effect of MeanT on dengue was observed, i.e., higher MeanT corresponding to higher rate of dengue incidence was only found in sub-period 1 when MeanT>27.8°C. In comparison, the effect of AH on dengue was more significant.

We also highlighted that, in the 9-year studied period, the dominant serotype has shifted every 3 years: Firstly, serotype 2 was the dominant one (sub-period 1: 2001–2003); then the dominant serotype shifted to serotype 1 in sub-period 2 (2004–2006); then in sub-period 3, it shifted back to serotype 2. Three key differences were observed in these three sub-periods:

- The predominant virus involved in each sub-period was distinctly different [34], [51];
- As a result, the level of relevant serotype-specific immunity in the population differs within each period;
- The control program shifted from a more reactive mode to a preventive mode with an increase of manpower from 250 in 2005 to 800 by 2012 [52].

It is interesting to note that the impact of AH on the risk of dengue was prominent for the first two sub-periods but not significant in sub-period 3. Sub-period 3 was also markedly different when MeanT was studied showing a reverse correlation when compared with sub period 1. The inconsistent pattern observed in sub-period 3 for both AH and MeanT suggests that one or more of the observed differences described above, could have played a role in modulating the correlation between dengue trends and the weather parameters. This demonstrates the need for studies of the correlation of infectious diseases with environmental parameters to take into consideration changes in control programs, circulating viruses and other epidemiological parameters.

Although in our study we have highlighted, based on our results that AH is an important weather indicator which impacts dengue incidence significantly, it does not mean that AH is the only weather factor to be considered for predicting dengue incidence. We had also carried out preliminary multivariate analysis to make further evaluation. The selection of weather factors to be included in the multivariate model is carried out according to QAIC: AH was first selected due to its minimum QAIC value among all candidate weather variables. Then, under the QAIC criterion, among the other weather factors, MeanT was the second one added to the model. After MeanT was selected representing temperature effect, both minimum and maximum temperatures were excluded from the variables selection procedure. The selection procedure continued among wind speed, relative humidity and rainfall. Then, lastly rainfall was the third one included in the model based on the QAIC criterion, i.e., an AH-MeanT-Rainfall model was constructed following our simplified selection approach. However, it was observed that the impact of AH on dengue incidence was similar irrespective of whether other weather factors were included during the modeling evaluation. We also used data in 2001–2008 to fit the two models (AH-MeanT-Rainfall model and AH model) and used the 2009 data for dengue prediction. The results (Mean Average Error) showed that the performance of the multivariate model (AH-MeanT-Rainfall model) was just slightly better than the AH model. This showed that AH can be a very useful weather factor for indicating dengue incidence trends. Furthermore, the use of a simple model with fewer variables would provide reference more clearly for policy makers in dengue surveillance operations. As this work focused on studying AH's impact on dengue incidence using our model, we believe that a more extensive research needs to be carried out to study the prediction models considering all the combination of AH and other available weather factors.

### Conclusions

Cross correlation analysis and DLNM modeling showed that AH was the best predictive weather factor among the weather factors studied. AH presented a more stable effect on indicating dengue incidence than MeanT did over the whole studied period as well as during sub-periods. A higher AH was associated with a higher dengue incidence. As such, AH could potentially be a better weather indicator for predicting dengue and assisting pro-active dengue prevention efforts in the future.

The shift of dominant serotypes and pre-emptive measures taken against dengue vectors since 2005 in Singapore may possibly explain the inconsistent weather-dengue patterns observed. As such, further studies are recommended to identify, evaluate and possibly include more diverse virological, immunological, entomological and public health factors into the dengue models.
